# In Situ Deformation Monitoring of 3D Woven Composite T-Profile Using MXene Nanoparticles

**DOI:** 10.3390/ma15082730

**Published:** 2022-04-07

**Authors:** Prasad Shimpi, Maria Omastova, Andrey Aniskevich, Daiva Zeleniakiene

**Affiliations:** 1 Department of Mechanical Engineering, Kaunas University of Technology, 51424 Kaunas, Lithuania; prasad.shimpi@ktu.edu; 2 Polymer Institute, Slovak Academy of Sciences, 84541 Bratislava, Slovakia; maria.omastova@savba.sk; 3 Institute for Mechanics of Materials, University of Latvia, LV-1004 Riga, Latvia; andrey.aniskevich@pmi.lv

**Keywords:** 3D woven composite, MXene, mechanical testing, T-profile

## Abstract

The aim of this study was to develop a process-efficient smart three-dimensional (3D) woven composite T-profile by depositing MXene nanoparticles at the junction for sensing damage and deformation at the junction. Such smart composites could find application in the online health monitoring of complex-shaped parts. The composites were manufactured by infusing epoxy resin in a single-layer fabric T-profile preform, woven in folded form on a dobby shuttle loom using 300 tex glass roving. The chemically etched Ti_3_C_2_T_z_ MXene nanoparticles were dispersed in deionised water and 10 layers were sprayed at the junction of the composite to form a conductive coating. The MXene-coated composite T-profile specimens were subjected to tensile and fatigue loading to study the electromechanical response of the MXene coating to applied displacement. The results showed that the MXene coating was able to sense the sample deformation till ultimate failure of the composite. The MXene coating was also able to effectively sense the tensile–tensile fatigue loading, carried out at 2000 cycles and 4000 cycles for a 50 N–0.5 Hz and a 100 N–1 Hz load–frequency combination, respectively, while being sensitive to the overall deformation of the composite. The smart complex-shaped composites developed in this work were capable of monitoring their health under tensile and fatigue loading in real time.

## 1. Introduction

Structural health monitoring (SHM) of fiber-reinforced composites is an important component of quality evaluation in aerospace, civil, and mechanical engineering structures. Researchers combine various scientific techniques to develop systems for sensing damage in the composite structures. These damage detection techniques can be broadly classified into two major categories, namely destructive and non-destructive testing (NDT). For small, bulk manufactured, and cheap components, the quality evaluation is carried out by destroying a sample out of the bulk quantity. However, parts that have complex designs and are expensive to manufacture are evaluated by NDT. Some of the NDT methods are ultrasonic [1], radiography [2], thermographic [3], acoustic [4], and shearography testing [5], etc. Although suitable for evaluation in a testing lab, these methods cannot provide real-time data of the damage during actual usage of the component.

To overcome the above-mentioned limitations, an approach of integrating sensor elements during various stages of composite manufacturing has been developed. Some of these techniques are integrating metallic wires, optical fibers [6,7], yarn coated with conductive dyes [8,9] etc., in the composite layup or in the fabric preform. However, sensor integration during the weaving process imparts damage to the sensor element and often results in improper functioning of the sensor. Placing sensor elements between fabric lamina during the composite layup induces defects in the final composite. To resolve this problem and enhance the sensing efficiency, conductive nanoparticles, such as MXene [10], carbon nanotube (CNT) [11,12], graphene [13], etc., are either dispersed in matrix [14], applied as coating on the composite [15], or deposited on the substrate surface by processes such as chemical vapor deposition [16,17] or nanoimprinting [18]. The mechanical deformation of the composite structure affects the electrical properties of these nanoparticles, which forms the basis of monitoring the structural health of the material.

The recently discovered MXene nanoparticles [19] are two-dimensional (2D) [20] structures of titanium carbide Ti_3_C_2_T_z_ with excellent mechanical and electrical properties. The T_z_ in the formula represents a functional group at the surface end, such as -O, -OH, and/or -F [19]. The reported tensile strength of MXene nanoparticles is 590 MPa (980 nm thick film) [21] and Young′s modulus ranges between 500 and 800 GPa from nanoindentation measurements [22]. MXene 2D nanoparticles are manufactured by selectively etching elements from its three-dimensional (3D) MAX phase [23]. The weak van der Waals force between the MXene nanoflakes makes it a more adaptable and sensitive material for strain-sensing applications [24]. Recent studies demonstrate high adhesion between MXene and epoxy resin [25]. The MXene nanoparticles can be homogeneously dispersed in matrix by techniques, such as melt blending [26], ex situ blending [27], and in situ polymerization [28].

The MXene layer conducts electricity by the virtue of quantum tunnelling and contact resistance [29]. Quantum tunnelling is the phenomenon in which the electrons can transfer between nano flake structures that have a gap in the order of 1 nm magnitude. Contact resistance is due to the overlapping of MXene over each other, thus providing a continuous path for electrical conductivity. The majority of research in the domain of SHM pertaining to MXene nanoparticles has been carried out on planar surfaces. However, in practical applications, the composite panels have complex shapes and geometries, according to the end use. One of the most extensively used shapes is the T-profile, which transfers loads between mutually perpendicular directions. The most common procedure to manufacture a T-profile shape in a composite is by laying up fabric plies in two opposing ‘L’ shapes on a rigid mould [30]. Although simple to manufacture, the laminated composite T-profiles are prone to premature failure and delamination when subjected to higher loads [31,32]. For applications in which higher structural stability is required, the laminated composite T-profiles are either z pinned/tufted [33], or the T-profile preform is 3D woven directly on a weaving machine [34,35]. Compared to all methods, the 3D woven composite T-profile exhibits higher load-bearing capacity, as continuous fibers are incorporated in the whole structure, forming a seamless preform that is resistant to delamination [36].

In laminated as well as 3D woven T-joint composites, the junction region connecting the base/web and the flange section acts as a critical failure zone, which has a higher probability for failure, in case the load exceeds the strength of the composite, thus attracting focus for reinforcement as well as health monitoring. Although common NDT techniques have been developed for the SHM of a T-joint, such as ultrasonic vibration [37], integrating piezoresistive layers [38], the integration of optical fibers based on fiber Bragg grating sensors [7], and electromechanical response of CNT [39], a research gap was found in the development of complex-shaped smart composites. The use of new 2D nanoparticles for this purpose could contribute to this progress. The aim of this research was to develop a smart, single-layer 3D woven composite T-profile, which would be easy to manufacture and could monitor its deformation and damage in real time using MXene nanoparticles. The composites were prepared by weaving a fabric T-profile and infusing it with epoxy resin. The MXene nanoparticles were sprayed on the junction of the composite T-profile. The feasibility of in situ deformation and damage sensing was investigated by subjecting the samples to tensile and fatigue loading, while monitoring the piezoresistive response of the MXene coating.

## 2. Materials and Methods

### 2.1. Preparation of MXenes

**Figure 1:**
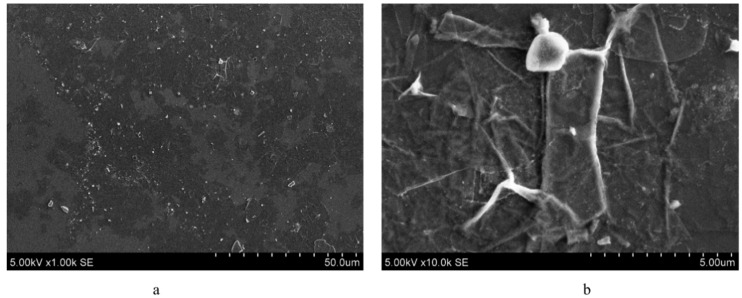
Scanning electron microscope analysis of MXene: (**a**) partially delaminated scattered MXene flakes; (**b**) single MXene flake.

Ti_3_C_2_T_z_ MXenes were prepared from Ti_3_AlC_2_ MAX phase (MRC, Kiev, Ukraine) by etching with hydrochloric acid (HCl, 37 wt.%, Merck, Darmstadt, Germany) and lithium fluoride (LiF, >99 wt.%, Sigma Aldrich, Munich, Germany). The MAX phase was added to the mixture of LiF/HCl and stirred for 24 h. A multilayer MXene sediment was formed at the bottom, which was further delaminated using 99 wt.% LiCl (Sigma Aldrich, Munich, Germany). The resulting solution was centrifuged at 3500 rpm 10–15 times and rinsed with deionized water until the pH of the supernatant reached 6.5. The measured concentration of MXenes in the supernatant was 0.335 mg/mL, which was further increased to 3.3 mg/mL by centrifuge to obtain conductivity in the order of 10^5^ Ω. Six layers of MXene were spread on a glass slide, gold coated, and subjected to a 15 keV electron beam under SEM. The partially delaminated MXene flakes with sizes ranging from 1 to 10 µm can be observed in Figure 1a. A close-up view of a single stack of MXene flakes with a size of 2.5 × 5 µm^2^ can be observed in Figure 1b. These images correspond well with the previous SEM analyses [10]. The average thickness of these MXene coatings, measured using an atomic force microscope, was 30 nm for 3 layers [25].

### 2.2. Weaving of T-Profile Preform and Composite Manufacturing

**Figure 2:**
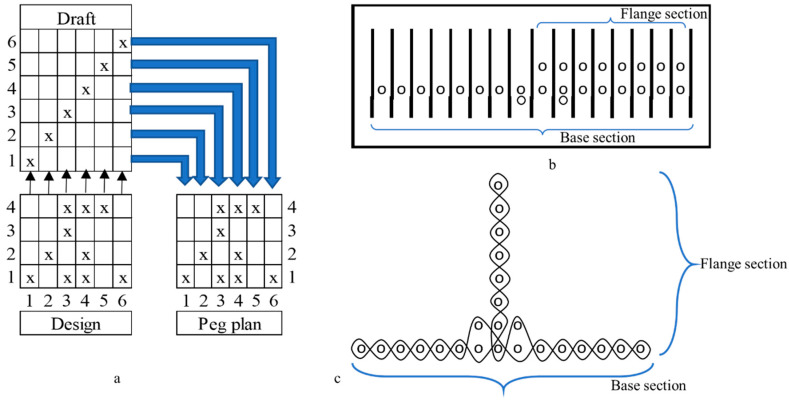
Weaving of T-profile: (**a**) weave design, draft and peg plan; (**b**) denting plan; (**c**) warp cross sectional view of T-profile.

The single-layer fabric T-profile preform was woven on an 8-head shaft dobby loom with shuttle insertion [40]. Glass rovings of 300 tex were threaded with a draft and denting plan, as shown in Figure 2a,b, respectively, such that the T-profile is woven in folded form. A plain weave was selected as a base, and the design, as shown in Figure 2a, was woven while keeping threads per cm (warp and weft) at a constant value of 8. As shown in Figure 2b,c, 2 additional rovings were threaded at the base section, near the junction of the T-profile, as reinforcement.

The bisphenol epoxy CR-122 and amine hardener supplied by Sika (Baar, Switzerland) were used as matrix and applied manually to the woven T-profile preform. In next step, the wet preform was placed in a metal mould and subjected to 101.3 kPa vacuum for 3 h. The preform was cured at room temperature for 24 h and post-cured in an oven at 80 °C for 5 h.

**Figure 3:**
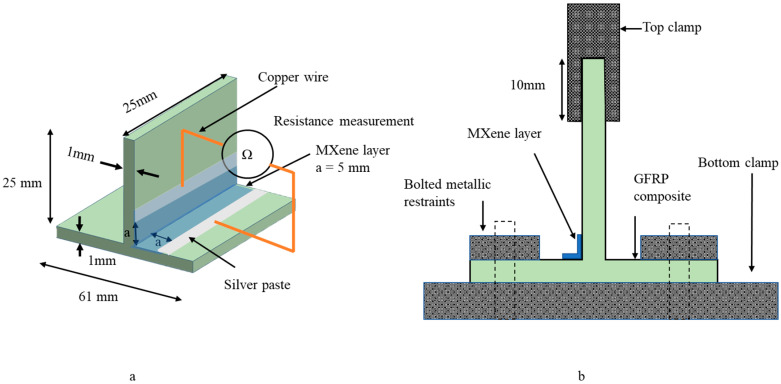
Three-dimensional woven composite T-profile: (**a**) specimen dimensions; (**b**) experimental setup for tensile and fatigue loading.

The composite T-profile with a thickness of 1 mm was treated with argon-oxygen plasma, cut into dimensions as shown in Figure 3a,b, and sprayed with 10 layers of MXene using a Sparmax HB-040 airbrush with a 0.4 mm diameter nozzle and a Sparmax DC-25X 2.07 bar compressor with 0.1 mL/s paint yield (Anest Iwata Sparmax Co., Taipei, Taiwan). Each layer was sprayed for 10 s at 15 cm distance from the preform in 250 mm^2^ area and dried at 60 °C using an air dryer.

### 2.3. Tensile Testing

Five specimens were cut to dimensions, as shown in Figure 3a,b, and tensile tested on an Instron ElectroPuls E10000T (Instron, Norwood, MA, USA) machine with a testing speed of 2 mm/min and a load cell capacity of 10 kN. The specimens were mounted as shown in Figure 3b. The base section of the composite was bolted to the bottom clamp with electric-insulating tape and the flange section of the composite was gripped in the top clamp with electric-insulating sandpaper to reduce slippage. Copper wires were soldered on the MXene coating and a silver paste was applied to reduce the contact resistance. The change in electrical resistance during tensile testing was measured using the two-probe method, shown in Figure 3a, via a Fluke 287 RMS multimeter (Fluke Corporation, Everett, WA, USA) with a Bluetooth data-logging system, whereas the contact resistance of 0.8 Ω was measured by taking the difference between the two-probe and four-probe methods of the multimeter contacts.

### 2.4. Fatigue Testing

Five composite specimens of dimensions, as shown in Figure 3a,b, were tested for tensile–tensile fatigue on a 10 kN load cell capacity Instron ElectroPuls E10000T (Instron, Norwood, MA, USA) machine with the parameters listed in Table 1. Each composite sample was tested for 6000 cycles, split into sets of 2000 and 4000 cycles, consecutively. The mounting of specimens on the machine and the measurement of change in electrical resistance, as shown in Figure 3a,b, were the same as those described in Section 2.3: Tensile Testing. The loading amplitude of the Instron machine was set in the elastic region of the sample. The measurement of cyclic displacement was functional only by internal loadcell of Instron machine; however, the probability of error in measuring displacement increases, due to slippage of samples at grip. To ensure the accuracy of displacement measurement, the loadcell was calibrated against an external optical strain sensor by tensile testing pristine bisphenol F epoxy dog-bone specimens according to ISO 527-2-5A. The difference in displacement measurement was 0.28%.

## 3. Results and Discussion

### 3.1. Tensile Testing Results and Dicussion

**Figure 4:**
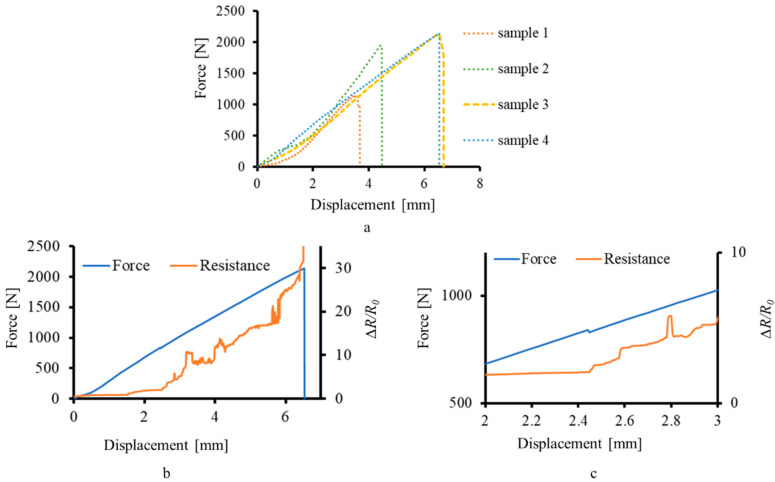
Real-time deformation monitoring of composite T-profile: (**a**) tensile testing of composite T-profile; (**b**) piezoresistive response of the MXene coating; (**c**) enlarged section of initial failure.

The graphs of force–displacement were plotted for four composite T-profile samples, as shown in Figure 4a. One sample was rejected out of five due to premature failure at the clip restrains. It was observed that the graph can be divided in two distinct zones, i.e., initial failure and final failure. Initial failure is usually detected as a drop in the tensile load values for the first time [40]. The curves of force and resistance show a statistically monotonic relationship, so its product-moment correlation coefficient was calculated by taking the ratio of covariance to standard deviation of force and absolute resistance datapoint values, for each of the four samples, presented in Table 2 along with the ultimate force of each sample. The range of the correlation coefficient is from −1 to 1, where −1 indicates a purely inverse relation and 1 indicates a purely direct relation between the datasets. As the behaviour of all the graphs is similar, the piezoresistive response of the MXene coating of one sample is shown in Figure 4b by plotting ∆*R/R*_0_ against force–displacement. The piezoresistive response of the MXene coating, corresponding to the deformation of the sample, matches well with the previous studies related to MXene-based SHM of composites [10,29].

As the sample elongated, the stress started to accumulate at the junction region of the T-profile. The resistance of the MXene coating started increasing, corresponding to the stress accumulation, as shown in Figure 4b. At 2.44 mm, as shown in Figure 4c, the initial failure occurred, which was detected as a disturbance in the resistance value of the MXene coating.

Between 2.44 mm and 6.54 mm displacement, the crack propagation started at the resin-rich area of the junction and propagated in the base section, leading to the catastrophic failure of the composite, thereby separating the flange and the base parts of the composite. It was observed that the crack did not spread near the restraints (clips at the base and clamp at the flange) and was restricted only at the base. The deformation due to stress accumulation till failure was detected as a steady increase in the resistance of the MXene coating, as shown in Figure 4b.

During tensile loading of the T-profile, the junction region acts as a point of change in the direction of load from the top clamp to the clip restraints and thus accumulates stress [30,41]. Initial failure of the composite occurs in the junction region, when the external applied force exceeds the tensile strength of the matrix. On further progression of the force, the yarn/matrix interface, and eventually the composite, fails. For the laminated composite, the main failure mode is delamination; however, for the 3D woven T-profile composite, the main failure mode observed is yarn/matrix damage at the junction [32].

On planar surfaces, the piezoresistive response of the MXene coating linearly corresponds to the applied strain [10]. Thus, the steady increase in the MXene coating resistance, as shown in Figure 4b, can be attributed to the combined elongation of the base and flange sections. It has been theoretically and experimentally reported that the crack formation initiates at the junction of the T-joint composite [31,32,35,40]. The MXene coating, being adhered to the composite [25], absorbs this fracture energy and starts to crack as well, showing spikes in the resistance values. At a macro level, the change in dimensions of the composite substrate causes the MXene coating to change its geometry and, in turn, the resistance, similar to a traditional strain gauge. At a micro level, the MXene coating consists of a network of individual MXene flakes, which absorb the fracture energy of the substrate and break, thus abruptly increasing the tunnelling and contact resistance.

### 3.2. Fatigue Testing Results and Discussion

**Figure 5:**
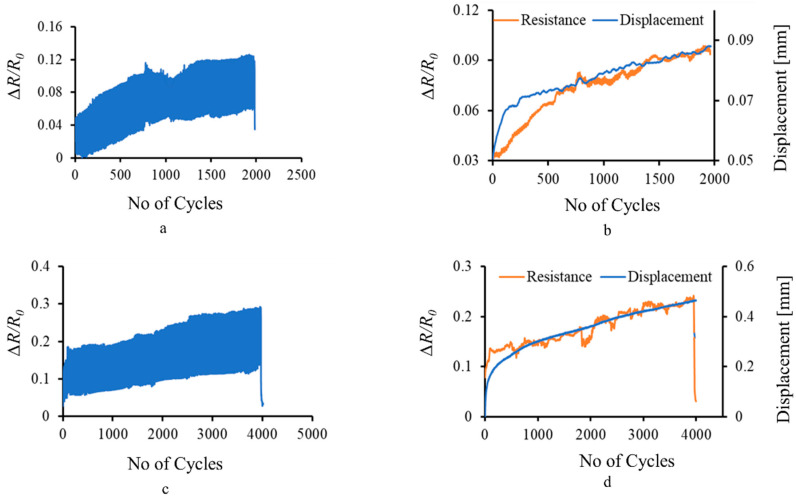
Real-time fatigue sensing of composite T-profile by MXene coating: (**a**) resistance measurement at 50 N–0.5 Hz; (**b**) sensing of displacement component during fatigue loading of 2000 cycles; (**c**) resistance measurement at 100 N–1 Hz; (**d**) sensing of displacement component during fatigue loading of 4000 cycles.

The graphs of Δ*R/R*_0_ versus number of cycles for one sample are plotted in Figure 5 and show the electromechanical response of the MXene, corresponding to fatigue loading in the elastic limit of the sample.

The change in resistance of the MXene coating corresponds to the cyclic stress imparted by the machine at 0.5 Hz frequency, as shown in Figure 5a. With the increase in the number of cycles, the load, displacement, and subsequent deformation also increased, which is sensed by the MXene coating as a cumulative increase in the resistance values, shown in Figure 5b, calculated by taking the moving average of resistance data points. Cracks and damage during the loading are sensed as an abrupt change in the resistance value at the same load as captured at 1000th cycle in Figure 5a. The 50 N load imparted by the machine was within the elastic limit of the composite T-profile sample; thus, minimum damage was imparted to the MXene coating as its resistance remained constant when measured before and after the 0.5 Hz frequency loading.

With an increase in the loading force to 100 N and the frequency to 1 Hz, stress accumulation and damage progression is rapid. The composite sample was stressed near the border of its elastic limit and thus accumulated slight damage. This was indicated by a 3.2% increase in the resistance values of the MXene coating before and after the test. However, the overall structural integrity of the MXene coating remained stable as it was still able to sense the cyclic stress and cumulative increase in the deformation, corresponding to the external displacement, as shown in Figure 5c,d, respectively. The electromechanical response of the MXene coating during cyclic loading at 0.5 Hz and 1 Hz obtained in this work corresponds well with the previous studies [10,29].

During fatigue loading of the composite sample, the MXene flakes undergo cyclic stress and relax, which causes a periodic increase and decrease in the resistance values, owing to tunnelling and contact resistance. However, during each cycle, the strain and the load on the sample increases, which accumulates stress at the junction of the composite. This causes a permanent increase in the resistance values of the MXene coating [29], as the individual MXene flakes drift apart and cannot return to their initial position. Owing to this mechanism, the resistance of the MXene coating directly corresponds to the cyclic stress, as well as the overall deformation, of the composite T-profile.

## 4. Conclusions

In this research work, a smart composite was developed by spraying MXene particles on a 3D woven composite T-profile. The MXene coating was able to sense the damage and deformation at the junction in real time under tensile and fatigue loading. The graphs of change in resistance versus force–displacement were plotted to study the electromechanical response of the MXene coating. The following conclusions can be derived from the study:

1. The concept of spraying MXene particles on the junction of the composite T-profile is efficient for real-time damage and deformation sensing of complex-shaped composites under tensile and fatigue loading. The partially delaminated MXene flakes of 3.3 mg/mL concentration were successful in sensing the deformation of the T-profile composite.
2. During tensile testing of the samples, the main failure zone observed was the junction region, indicating its critical nature and the need for health monitoring. The MXene coating sprayed at the junction was able to sense the initial failure at 2.44 mm displacement as a spike in the resistance values, as well as the total deformation till failure as a steady increase in the resistance values.
3. Damage during the fatigue loading was sensed as an abrupt change in the resistance value for same loading conditions. During fatigue loading at 50 N and 100 N, the MXene coating was stable and captured the cyclic stress at 0.5 Hz and 1 Hz, respectively. At a 50 N–0.5 Hz load-frequency combination, there was no damage to the MXene coating and, thus, the resistance values remained stable. However, at a 100 N–1 Hz load–frequency, there was a permanent increase of 3.2% in the resistance of the MXene coating, which indicates a threshold level of damage initiation.
4. The smart composites in this study are easy to manufacture and cost-efficient; however, the sensing function is only limited for deformation monitoring. In the future scope of work, the piezoresistive response of the MXene nanoparticles can be calibrated against conventional strain gauges mounted on the base and the flange sections of the T-profile. The data thus obtained can be processed to determine stress at various zones in the complex-shaped composites.
5. The concept of this research work can be applied to monitor large complex-shaped components that are expensive and require monitoring at critical junctions. The dimensional tolerances of such components are strict and, thus, the incorporation of traditional strain gauges and external sensor elements is difficult. In such scenarios, the conductive coating of MXene nanoparticles in a small area can be an effective solution.

## Figures and Tables

**Table 1:** Fatigue testing parameters.

Parameter	Values	
No. of cycles	2000	4000
Frequency (Hz)	0.5	1
Load (N)	50	100

**Table 2:** Correlation coefficient and ultimate tensile force.

Parameter	Sample 1	Sample 2	Sample 3	Sample 4
Correlation coefficient	0.70	0.68	0.71	0.68
Ultimate tensile force (N)	1150	2138.5	2140.2	1918.4

## Data Availability

Not applicable.

## References

[1] Jolly M.R., Prabhakar A., Sturzu B., Hollstein K., Singh R., Thomas S., Foote P., Shaw A. (2015). Review of Non-destructive Testing (NDT) Techniques and their Applicability to Thick Walled Composites. Procedia CIRP.

[2] Dwivedi S.K., Vishwakarma M., Soni A. (2018). Advances and Researches on Non Destructive Testing: A Review. Mater. Today Proc..

[3] Ciampa F., Mahmoodi P., Pinto F., Meo M. (2018). Recent Advances in Active Infrared Thermography for Non-Destructive Testing of Aerospace Components. Sensors.

[4] Crivelli D., Guagliano M., Eaton M., Pearson M., Al-Jumaili S., Holford K., Pullin R. (2015). Localisation and identification of fatigue matrix cracking and delamination in a carbon fibre panel by acoustic emission. Compos. Part B Eng..

[5] Gholizadeh S. (2016). A review of non-destructive testing methods of composite materials. Procedia Struct. Integr..

[6] Giurgiutiu V. (2015). Structural health monitoring (SHM) of aerospace composites. Polymer Composites in the Aerospace Industry.

[7] Hisada S., Minakuchi S., Takeda N. (2021). In-Situ Strain Monitoring in Deltoid of Composite T-joints using Optical Fiber. Struct. Health Monit..

[8] Nauman S., Cristian I., Koncar V. (2012). Intelligent carbon fibre composite based on 3D-interlock woven reinforcement. Text. Res. J..

[9] Ning F., He G., Sheng C., He H., Wang J., Zhou R., Ning X. (2021). Yarn on yarn abrasion performance of high modulus polyethylene fiber improved by graphene/polyurethane composites coating. J. Eng. Fibers Fabr..

[10] Monastyreckis G., Stepura A., Soyka Y., Maltanava H., Poznyak S., Omastová M., Aniskevich A., Zeleniakiene D. (2021). Strain Sensing Coatings for Large Composite Structures Based on 2D MXene Nanoparticles. Sensors.

[11] Shimpi P., Aniskevich A., Zeleniakiene D. (2021). Improved method of manufacturing carbon nanotube infused multifunctional 3D woven composites. J. Compos. Mater..

[12] Stankevich S., Bulderberga O., Tarasovs S., Zeleniakiene D., Omastova M., Aniskevich A. (2021). Electrical Conductivity of Glass Fiber-Reinforced Plastic with Nanomodified Matrix for Damage Diagnostic. Materials.

[13] Tang L.-C., Wan Y.-J., Yan D., Pei Y.-B., Zhao L., Li Y.-B., Wu L.-B., Jiang J.-X., Lai G.-Q. (2013). The effect of graphene dispersion on the mechanical properties of graphene/epoxy composites. Carbon.

[14] Forcellese A., Simoncini M., Vita A., Giovannelli A., Leonardi L. (2020). Performance analysis of MWCNT/Epoxy composites produced by CRTM. J. Mater. Process. Technol..

[15] Khan T.A., Nauman S., Asfar Z., Nasir M.A., Khan Z. (2018). Screen-printed nanocomposite sensors for online in situ structural health monitoring. J. Thermoplast. Compos. Mater..

[16] Turgut F., Koycu A., Neje G., Behera B., Yenigun E.O., Cebeci H. Hierarchical CNTs Grown Multifunctional 3D Woven Composite Beams for Aerospace Applications. Proceedings of the AIAA Scitech 2020 Forum.

[17] Handschuh-Wang S., Wang T., Tang Y. (2021). Ultrathin Diamond Nanofilms—Development, Challenges, and Applications. Small.

[18] Xiao C., Zhao Y., Zhou W. (2020). Nanoimprinted conducting nanopillar arrays made of MWCNT/polymer nanocomposites: A study by electrochemical impedance spectroscopy. Nanoscale Adv..

[19] Gong K., Zhou K., Qian X., Shi C., Yu B. (2021). MXene as emerging nanofillers for high-performance polymer composites: A review. Compos. Part B Eng..

[20] Zhu H., Zhu J., Zhang Z., Zhao R. (2021). Crossover from Linear Chains to a Honeycomb Network for the Nucleation of Hexagonal Boron Nitride Grown on the Ni(111) Surface. J. Phys. Chem. C.

[21] Zhang J., Kong N., Uzun S., Levitt A., Seyedin S., Lynch P.A., Qin S., Han M., Yang W., Liu J. (2020). Scalable Manufacturing of Free-Standing, Strong Ti 3 C 2 T x MXene Films with Outstanding Conductivity. Adv. Mater..

[22] Plummer G., Anasori B., Gogotsi Y., Tucker G.J. (2018). Nanoindentation of monolayer Ti C T MXenes via atomistic simulations: The role of composition and defects on strength. Comput. Mater. Sci..

[23] Ng V.M.H., Huang H., Zhou K., Lee P.S., Que W., Xu J.Z., Kong L.B. (2016). Recent progress in layered transition metal carbides and/or nitrides (MXenes) and their composites: Synthesis and applications. J. Mater. Chem. A.

[24] Liu J., Tang J., Gooding J.J. (2012). Strategies for chemical modification of graphene and applications of chemically modified graphene. J. Mater. Chem..

[25] Zukiene K., Monastyreckis G., Kilikevicius S., Procházka M., Micusik M., Omastová M., Aniskevich A., Zeleniakiene D. (2020). Wettability of MXene and its interfacial adhesion with epoxy resin. Mater. Chem. Phys..

[26] Shi Y., Liu C., Liu L., Fu L., Yu B., Lv Y., Yang F., Song P. (2019). Strengthening, toughing and thermally stable ultra-thin MXene nanosheets/polypropylene nanocomposites via nanoconfinement. Chem. Eng. J..

[27] Gao L., Li C., Huang W., Mei S., Lin H., Ou Q., Zhang Y., Guo J., Zhang F., Xu S. (2020). MXene/Polymer Membranes: Synthesis, Properties, and Emerging Applications. Chem. Mater..

[28] Hai Y., Jiang S., Zhou C., Sun P., Huang Y., Niu S. (2020). Fire-safe unsaturated polyester resin nanocomposites based on MAX and MXene: A comparative investigation of their properties and mechanism of fire retardancy. Dalton Trans..

[29] Wang X., Lu J., Lu S., Li B., Zhang L., Ma C., Ma K., Lin L., Jiang X., Yang B. (2021). Health monitoring of repaired composite structure using MXene sensor. Compos. Commun..

[30] Stickler P.B., Ramulu M. (2006). Experimental study of composite T-joints under tensile and shear loading. Adv. Compos. Mater..

[31] Bai J.-B., Dong C., Xiong J.-J., Luo C.-Y., Chen D. (2018). Progressive damage behaviour of RTM-made composite T-joint under tensile loading. Compos. Part B Eng..

[32] Yan S., Zeng X., Long A. (2018). Experimental assessment of the mechanical behaviour of 3D woven composite T-joints. Compos. Part B Eng..

[33] Bigaud J., Aboura Z., Martins A., Verger S. (2018). Analysis of the mechanical behavior of composite T-joints reinforced by one side stitching. Compos. Struct..

[34] Sugun B., Shimpi P., Sugun B.S. (2021). Orthogonal weaving. Practical Approach to 3D Weaving.

[35] Sugun B., Sandeep D. (2016). Development of single-layer 3D ‘T’ profile with fillet for composite ‘T’ joints. J. Ind. Text..

[36] Wang Y., Soutis C. (2016). Fatigue Behaviour of Composite T-Joints in Wind Turbine Blade Applications. Appl. Compos. Mater..

[37] Ooijevaar T., Loendersloot R., Warnet L., de Boer A., Akkerman R. (2010). Vibration based Structural Health Monitoring of a composite T-beam. Compos. Struct..

[38] Panda S., Mishra P. (2021). Damage Propagation Prediction of Adhesion Failure in Composite T-joint Structure and Improvement using PZT Patch. Sci. Iran..

[39] Wan Y., Hu W., Yang B., Zhao X., Xian G., Yuan Y., He L., Liu C., Deng J. (2020). On-line tensile damage monitoring of WGF/epoxy T-joint by the embedded MWCNT@WGF sensor. Compos. Commun..

[40] Verma K.K., Sandeep D.N., Sugun B.S., Athimoolaganesh S. Novel Design of Cocured Composite ‘T’ Joints with Integrally Woven 3D Inserts. Proceedings of the 5th World Conference on 3D Fabrics and Their Applications.

[41] Yan S., Zeng X., Brown L., Long A. (2017). Geometric modeling of 3D woven preforms in composite T-joints. Text. Res. J..

